# The mitochondrial genome of the terrestrial carnivorous plant *Utricularia reniformis* (Lentibulariaceae): Structure, comparative analysis and evolutionary landmarks

**DOI:** 10.1371/journal.pone.0180484

**Published:** 2017-07-19

**Authors:** Saura R. Silva, Danillo O. Alvarenga, Yani Aranguren, Helen A. Penha, Camila C. Fernandes, Daniel G. Pinheiro, Marcos T. Oliveira, Todd P. Michael, Vitor F. O. Miranda, Alessandro M. Varani

**Affiliations:** 1 Departamento de Botânica, Instituto de Biociências, Universidade Estadual Paulista (UNESP), Botucatu, São Paulo, Brazil; 2 Departamento de Tecnologia, Faculdade de Ciências Agrárias e Veterinárias, Universidade Estadual Paulista (Unesp), Jaboticabal, São Paulo, Brazil; 3 Departamento de Biologia Aplicada à Agropecuária, Faculdade de Ciências Agrárias e Veterinárias, Universidade Estadual Paulista (Unesp), Jaboticabal, São Paulo, Brazil; 4 Computational Genomics, Ibis Bioscience, Carlsbad, CA, United States of America; University of Western Sydney, AUSTRALIA

## Abstract

The carnivorous plants of the family Lentibulariaceae have attained recent attention not only because of their interesting lifestyle, but also because of their dynamic nuclear genome size. Lentibulariaceae genomes span an order of magnitude and include species with the smallest genomes in angiosperms, making them a powerful system to study the mechanisms of genome expansion and contraction. However, little is known about mitochondrial DNA (mtDNA) sequences of this family, and the evolutionary forces that shape this organellar genome. Here we report the sequencing and assembly of the complete mtDNA from the endemic terrestrial Brazilian species *Utricularia reniformis*. The 857,234bp master circle mitochondrial genome encodes 70 transcriptionaly active genes (42 protein-coding, 25 tRNAs and 3 rRNAs), covering up to 7% of the mtDNA. A *ltrA*-like protein related to splicing and mobility and a LAGLIDADG homing endonuclease have been identified in intronic regions, suggesting particular mechanisms of genome maintenance. RNA-seq analysis identified properties with putative diverse and important roles in genome regulation and evolution: 1) 672kbp (78%) of the mtDNA is covered by full-length reads; 2) most of the 243kbp intergenic regions exhibit transcripts; and 3) at least 69 novel RNA editing sites in the protein-coding genes. Additional genomic features are hypothetical ORFs (48%), chloroplast insertions, including truncated plastid genes that have been lost from the chloroplast DNA (5%), repeats (5%), relics of transposable elements mostly related to LTR retrotransposons (5%), and truncated mitovirus sequences (0.4%). Phylogenetic analysis based on 32 different Lamiales mitochondrial genomes corroborate that Lentibulariaceae is a monophyletic group. In summary, the *U*. *reniformis* mtDNA represents the eighth largest plant mtDNA described to date, shedding light on the genomic trends and evolutionary characteristics and phylogenetic history of the family Lentibulariaceae.

## Introduction

Carnivorous plants have highly specialized morphological and physiological features adapted to uptake nutrients from captured prey as an alternative source of nutrients, thus supplementing the deficiency that comes from oligotrophic soils [1,2]. These plants are mostly found in low vegetation, from sandy to granitic soils, in water bodies and even in small flooded films, which are harsh conditions for most plants, but well tolerated by carnivorous plants [3,4]. This wide range of habitats is accompanied by a number of life forms and nutrient uptake mechanisms associated with the prey trap itself and with the trap microbiome [5–10] For the family Lentibulariaceae, their peculiar morphology encompasses structures that do not always follow the traditional morphological classification, with well-defined leaf, stem and root organs [11]. For example, species from the genera *Utricularia* and *Genlisea* absorb nutrients through their leaves, phylloclades and/or utricles (traps) and lack roots [3,12,13].

Although the genetic architecture of several carnivorous plants is yet to be elucidated, the aquatic bladderwort *Utricularia gibba* has recently been considered as an interesting model plant, since it represents a specialized group from the family Lentibulariaceae, with species that have the smallest nuclear genomes among angiosperms known to date at 101Mbp [14]. Interestingly, its organellar genomes maintain typical sizes and features, such as gene content, genomic recombination, insertion of foreign DNA, and RNA editing [15], which are shared with the chloroplast DNA (cpDNA) of the terrestrial *U*. *reniformis*, although loss and pseudogenization of the NAD(P)H-dehydrogenase genes have been observed in this case [16].

*U*. *reniformis* A.St.-Hil. is endemic to Brazil, growing as a terrestrial plant in wet grasslands, and in contrast to *U*. *gibba*, exhibits a larger and polyploid genome with high levels of heterozygosity [17]. However, mitochondrial DNA (mtDNA) sequence information is scarce for carnivorous plants, including *Utricularia*, most likely due to the complex features commonly found in this angiosperm mitochondrial genome. Recently, the partial mtDNA of *U*. *gibba* was deciphered by third-generation genome sequencing approaches, indicating a genome size of 283,823 bp [14], but no further analyses were performed. Nonetheless, it has been proposed that *Utricularia* has significantly higher nucleotide substitution rates in several mtDNA *loci* [18,19], and that this may be related to the increased respiration rates and, consequently, increased production of reactive oxygen species (ROS) which accompanies its carnivorous habit [5]. This phenomenon could have contributed to the rapid morphological evolution of the terrestrial, epiphytic, reophytic, and aquatic forms observed for this group, since the high abundance of ROS can lead to an accumulation of nucleotide substitutions in all genomic compartments (mitochondrial, chloroplast and nuclear) [19,20]. Therefore, besides the conserved processes of plant ATP production and synthesis of amino acids, vitamins, and lipids, mitochondrial function in the Lentibulariaceae species may also have significantly influenced the genome evolution, maybe contributing to the diverse bodyplan organizations and habitat adaptations.

Herein, we describe the sequencing and assembly of the first complete mtDNA sequence from the species *Utricularia reniformis*, using a combination of paired-end and mate-pair short read sequences. Annotation, comparative genomics and phylogenomics indicated that *U*. *reniformis* mtDNA retains common features often found in angiosperm mtDNA, providing useful insights into the genomic trends and evolutionary characteristics and phylogenetic history of the family Lentibulariaceae.

## Material and methods

### Plant sampling

*U*. *reniformis* samples were collected in the fall of 2015 in the Serra do Mar Atlantic Forest reserve (Geographic Location: 23°31'315"S– 45°53'53"O, 781m a.s.l), located in the Municipality of Salesópolis, State of São Paulo, Brazil, and deposited in the Herbarium JABU of the São Paulo State University (voucher V.F.O de Miranda et al., 1725). No permission for collecting was necessary, as the sample was not collected in protected areas and *U*. *reniformis* is not a threatened species according to the global IUCN (The IUCN Red List of Threatened Species - http://www.iucnredlist.org) and the Brazilian List of Threatened Plant Species.

### Mitochondrial sequencing and assembly

Total DNA was extracted following the QIAGEN DNeasy Plant Maxi Kit extraction protocol (QIAGEN). Whole-genome shotgun sequencing was performed using the Illumina MiSeq platform with a paired-ends (PE) library of 2x300bp and an average insert size of ~600 bp. Library construction followed the Illumina Nextera XT Preparation Guide (Illumina, USA). A total of 40M PE reads were generated. Furthermore, an additional set of 160M mate-paired (MP) reads (2x100 bp) with an average insert size of ~3,500 bp (fragment sizes varying from 1kbp to up to 9kbp) were generated using Illumina HiScanSQ platform. Low quality sequences (Phred scores < 24), contaminants, adapters, and sequences with less than 50bp were removed using Platanus_trim [21], leaving 36M (PE) and 150M (MP) high quality reads for the mtDNA assembly.

The assembly was conducted in seven steps, described below:

1. Trimmed PE and MP reads with full-length matches to the *U*. *reniformis* chloroplast genome [16] were discarded with bowtie2 v2.2.9 using—*very-sensitive* and—*end-to-end* parameters [22];
2. Filtered PE reads were assembled with CLC Genomics Workbench v9 (QIAGEN Aarhus, Denmark– http://www.clcbio.com), and the coverage of each assembled sequence was estimated for identification of abnormal coverage peaks, allowing the identification of potential mitochondrial-derived regions. It is expected that the coverage depth of a plant mitochondrial genome assembly is relatively constant across the genome, with peaks corresponding to plastid and duplicated regions;
3. Potential mitochondrial contigs were baited by mapping against plant mitochondrial genes commonly found in angiosperms [23] with BLAST v2.2.26 [24];
4. PE and MP reads generated from the (b) step were mapped back to the retrieved contigs from the (c) step with bowtie2. This resulted in a set of high-quality and filtered mitochondrial reads;
5. To avoid misassemblies and incorrect contig linking due to the presence of repeats or dynamic and multipartite structures commonly found in the angiosperm mitochondrial genomes [25], only the PE reads were assembled with SPAdes v3.9.0 [26] with default parameters, and the assembly graph was inspected with Bandage [27]. The MP reads were used in the next steps for resolution of repeats and master circle assembly;
6. Each assembled contig from the previous step was extended independently by iterative (mapping) assembly with MITObim v1.9 [28], allowing the identification of repeated sequences and possible connections between the contigs. During this process, the joining of contigs and scaffold construction were based on sequence similarity of terminal regions of each contig with a minimum overlap of 100 bp and >99% identity;
7. To validate the joining of contigs and for repeat resolution, the MP reads were mapped back to the extended sequences from the (f) step with bowtie2, and the assembly paths were inspected using a custom Perl program. To guarantee the correct assembly of each long repeated sequence (>300bp), three approaches were used: (1) depth-coverage analysis, in which a higher coverage is expected in the repeated regions than is observed in non-repeated regions; (2) at least 50 PE and 10 MP reads supporting the anchoring of each repeat to their respective genome location; (3) individual mapping and assembly of each repeated sequence to their respective anchoring borders, where the assembled repeats had to be concordant with at least two different assembly software (SPAdes and Platanus 1.2.4). This method ensured a higher confidence assembly of repeats longer than 300bp.

The master circle was manually constructed by analyzing the longest assembly path, composed of all the contigs including the repeats, and with support of MP read mapping across the entire sequence with the use of the CLC Genomics Workbench v9 (minimum of 10 different MP reads for each contig joining). The remaining gaps were closed with GapCloser v1.12 from SOAPdenovo2 package v2.04 [29]. The average coverage depth was estimated with bowtie2 with—*very-sensitive* and—*end-to-end* parameters and samtools *depth* [30].

### Annotation and analysis of the mitochondrial genome

The mtDNA was annotated using MITOFY (Annotation of Plant Mitochondrial Genomes) [31] coupled with Prodigal v2.6.2 [32] using the standard genetic code, ARAGORN [33], and BLAST for additional gene location refinements. Corrections of start and stop codons, intron acceptor and donor sites, and annotation curation were performed with Artemis genome browser 16.0.0 [34]. For gene assignments, the Blast2GO tool [35] was used. Potential plastid-like sequences were identified with BLASTn and DOGMA (Dual Organellar GenoMe Annotator) [36]. Identification of potential mitovirus-derived sequences was carried out by tBLASTn searches against the available mitovirus RNA-directed RNA polymerase protein sequences from the Uniprot database [37]. Putative transposable elements were identified with RepeatMasker open-4.0.5 (http://www.repeatmasker.org), using the Viridiplantae dataset from the Repbase database version 20150807 [38]. Group I and II introns were detected with the RNAweasel tool [39]. Potential truncated pseudogenes were defined by BLAST comparative analysis with the use of at least one of the following criteria:

1. presence of at least one stop codon in-frame within the predicted coding region;
2. absence of start and/or stop-codon;
3. frameshift;
4. lacking of at least 20% of the coding region when compared to the respective coding region of closely related species.

A circular gene map was drawn with OGDRAW (OrganellarGenome DRAW) [40]. Regions repeated within the mitochondrial genome, and with high similarity between *Utricularia gibba* draft mtDNA and the *U*. *reniformis* chloroplast genome were detected with BLASTn with the following parameters: e-value cutoff of 1^−10^ and at least 90% sequence identity. Comparative circular maps were generated with ClicO FS [41] and Circus v0.64 [42].

The annotated sequences and raw reads of the *Utricularia reniformis* mitochondrial genome have been deposited in the GenBank database under accession numbers [GenBank: KY774314, SRX2646130 and SRX2646131] (BioProject PRJNA290588).

### Phylogenetic analyses

The concatenated alignment of the *atp1*, *cox1*, *matR*, *nad5*, *rps3* genes from 32 different species from the Lamiales order (S1 Table) was performed using MAFFT v7.123b [43] with default parameters. For the probabilistic analysis, the best evolutionary models (best-fit) were tested using ModelTest 3.7 [44]. Thus, the best-fit DNA model was evaluated for the combined dataset with the corrected Akaike information criterion [45,46]. Maximum likelihood (ML) and Bayesian inferences were performed to estimate the phylogenetic hypothesis for the dataset. ML analyses were run with RAxML v8 [47]. For the ML analyses the GTR+GAMMA+I model was selected with ModelTest, and 10,000 bootstrap pseudoreplicates were applied. Bayesian inferences were performed with MrBayes software version 3.2.5 [48] with 5 x 10^5^ generations sampled for each 100 generations, using the default parameters. For each analysis, two runs (nruns = 2) with four chains (nchains = 4) were performed beginning from random trees. Initial samples were discarded after reaching stationary (estimated at 25% of the trees). Cladograms were drawn with TreeGraph2 v2.11.1–654 beta [49].

### RNA-seq and RNA-edit analyses

Three different organs from plants from the same natural population in field were frozen in liquid N_2_ and used for RNA-seq analysis: fresh leaves, stolons and utricles. The tissues were pooled in three replicates and total RNA was extracted using the PureLink RNA Mini Kit (Thermo Fisher Scientific), according to the manufacturer protocol. DNase I (Thermo Fisher Scientific) was used to remove any genomic DNA contamination. The extracted RNA was evaluated using an Agilent 2100 Bioanalyzer (Agilent Technologies) and a Qubit 2.0 Fluorometer (Invitrogen). Only samples having an RNA integrity number (RIN) ≥ 7.0 were used for the sequencing. cDNA libraries were sequenced on the Ion Proton system (Life Technologies) generating 180M reads with an average read length of 200bp. Low quality sequences (Phred < 20), bacterial contaminants, adapters, and sequences with less than 20bp were removed using prinseq_lite v0.20.4 [50].

To distinguish potential nuclear/plastid-like transcripts from potential authentic mitochondrial transcripts, two different approaches were used. First, filtered RNA reads were mapped back to the *Utricularia reniformis* mtDNA with bowtie2, with the—*very-sensitive* and—*end-to-end* parameters, and only full-length matches were considered. Second, the selected reads from the first step were mapped to traditional mtDNA genes with CLC Genomics Workbench v9 using the following parameters: mismatch cost of 3, insertion cost of 3, deletion cost of 3, minimal alignment coverage of 90% (Length fraction) and similarity fraction of >98%. The RNA-seq read mapping and transcription abundance were evaluated by RPKM (Reads Per Kilobase Million) normalization, whereas only unique read mappings were considered. In addition, intronic regions of intron-containing genes were also considered for the identification of spliced exons.

RNA-editing analyses based on the transcriptome data were carried out according to a previously proposed methodology [16]. In addition, the PREP-Mt tool [51] was used with default parameters to predict additional RNA editing sites. The mt RNA-seq reads used in this study have been deposited in the GenBank database under accession number [GenBank: SRX2646180] (BioProject PRJNA290588).

## Results

### Assembly of the *U*. *reniformis* mitochondrial genome

A total of 1,787,363 and 178,224 high-quality PE and MP mitochondrial reads were filtered from the raw reads generated by the Illumina MiSeq and HiScanSQ platforms, respectively. Approximately 830kbp (excluding repeated regions) were assembled into 7 contigs, with N50 length of 230kbp. The assembly graph analysis supports a complex scenario where 13 nodes, 8 edges, and 5 connected components lead to 14 dead ends, representing 53.85% of the assembled genome (Table 1 and S1 Fig). To investigate the dead ends and to determine the master circle molecule, the assembled contigs were independently extended by iterative read mapping. This analysis, together with the PE and MP read mapping, provided several contig connections that allowed the construction of different, inter-connected scaffolds, suggesting that a diverse set of alternative structures may occur *in vivo*. For instance, distinct smaller circular, short linear and branched structures were detected depending on the path taken to complete assembly. Interestingly, two repeated regions, spanning ~25kbp (LIR; long inverted repeat) and 3.2kbp (SDR; small direct repeat), consistently appeared during the contig extension process. Individual assembly of each repeat to their flanking borders resulted in their anchoring in the mtDNA sequence, supporting that these repeats are in fact present. In addition, the MP read mapping analysis supported the LIR assembly, with both flanking borders completely and concordantly anchored to the assembly (S2 Fig). As expected, these repeated regions showed a constant and higher than the estimated average coverage on non-repeated regions (Table 1). However, the coverage is not twice as high, which can be explained by our method to bait and assemble the mitochondrial genome, the sequencing technology bias, and, as expected for plant mtDNA, the presence of several and different alternative linear and circular structures, that do not exist in equal stoichiometric frequencies, leading to a biased coverage estimation analysis. These findings strongly suggest that, as observed for other plants [52,53], the *U*. *reniformis* mtDNA is composed of multipartite structures, with repeat-mediated recombination processes acting as key drivers of structural variation.

**Table 1:** Assembly summary statistics and validation of *Utricularia reniformis* mtDNA master circle (MC) genome.

Number of mtDNA-related paired-end reads (2x300bp ~600bp)	1,787,363
Number of mtDNA-related mate-paired reads (2x100bp ~3kbp)	178,224
Total assembled size (excluding repeats > 500bp)(bp)	831,638bp
PE assembly statistics	
- Nodes	13
- Edges	8
- Dead ends	14 (53.85%)
- Connected components	5
- Contigs	7
- Longest contig size	335,336bp
- Shortest contig size	40,798bp
- Average contig size	118,634bp
- N50	230,405
- L50	2
Master circle size (bp)	857,234
Master circle size GC%	43,98
- Average coverage	
- Paired-end	956x (st.dev. 265)
- Mate-pairs	40.98x (st.dev. 20.9)
- Long Repeat (LR) coverage (25kb)	1,342 (st.dev. 200)
- Small Repeat (SR) coverage (3.2kb)	1,201 (st. dev. 240)
- Mapped reads in pairs	
-paired-end reads	1,762,585 (98.61%)
-mate-paired reads	177,316 (99.49%)
- uncalled MC bases (Ns)	9 (0.0010%)
RNAseq mapping	
- Total Number of Reads (single-end ~200bp)	1,213,898
- Mitochondrial genome covered (bp)	672,561 (78.45%)
- Average coverage	83.9x (st. dev. 566.4)

Manual examination of the MP read mapping identified the most parsimonious master circle (MC) structure containing all the mtDNA related sequences. This resulted in a MC of 857kbp, with a GC content of 43.98% and an average coverage of 956X (+/- 265), with few uncalled bases (0.0010%). In addition, a total of 1,762,585 (98.61%) and 177,316 (99.49%) PE and MP reads, respectively, were mapped in pairs at the expected distance and orientation across the entire MC genome (Table 1), and no abnormal variation of coverage were observed in the non-repeated regions, thus supporting the MC assembly. According to the GenBank Organelles Database (https://www.ncbi.nlm.nih.gov/genome/organelle), among the 256 plant mtDNA genomes completely sequenced to date, the *U*. *reniformis* mtDNA represents the eighth largest, and the biggest one in the Lamiales order. The largest mitochondrial genome belongs to *Silene noctiflora* and *S*. *conica* (Caryophyllales), exhibiting a multichromosomal mtDNA genome, with ~59 and ~128 chromosomes ranging from 6.7 to 11.3Mb, respectively [54], followed by *Corchorus capsularis* and *C*. *olitorius* (Malvales) with 1,9Mbp and 1,8Mbp, respectively, *Cucumis sativus* and *C*. *pepo* (Cucurbitales) with 1,6Mbp and 982kbp, respectively, and *Welwitschia mirabilis* (Welwitschiales, the tree tumbo gymnosperm) with 978kbp.

### The *U*. *reniformis* mtDNA content and organization

**Fig 1:**
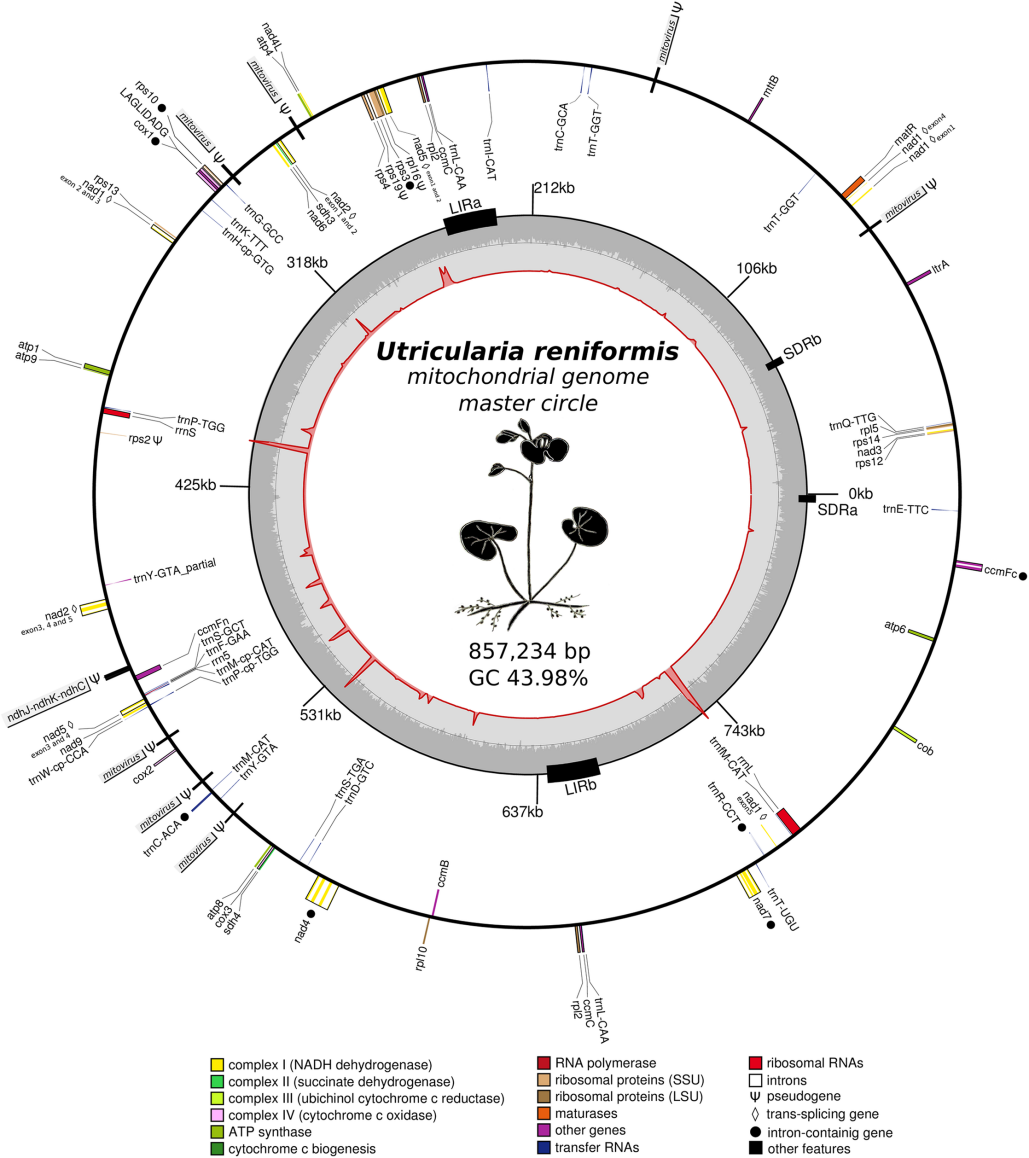
Genomic map of the *Utricularia reniformis* mtDNA genome. The inner red circle illustrates the transcript depth of coverage for *U*. *reniformis* mtDNA, whereas the peaks represent the most covered regions by RNAseq reads. The second level circle in gray scale represents the GC% distribution across the mtDNA. The Large inverted repeats (LIR) and Small direct repeats (SDR) are shown as black boxes. The mitochondrial genes are shown in the outer circle, whereas genes shown on the outside of the map are transcribed clockwise, and the genes on the inside are transcribed counter-clockwise. Genes are color coded by their function in the legend, whereas partial mitovirus derived sequences are shown in gray boxes. The *ndhJ*-*ndhK*-*ndhC* loci which is deleted from the *U*. *reniformis* plastid genome is shown in the gray box.

*Utricularia reniformis* presents a typical plant mitochondrial genome (Fig 1 and Table 2). The mtDNA encodes 70 mitochondrial genes, including 42 protein-coding, 25 tRNAs and 3 rRNAs, and an additional truncated copy of *rrna5* (Table 3). Two identical copies of the genes *ccmC*, *rpl2* and *trnL*-CAA were identified in the LIR regions, and two copies of the *trnT*-GGT gene were found in the SDR regions (Fig 1). As observed in other angiosperm mtDNA [23], the *U*. *reniformis* mtDNA does not contain a complete tRNA set, indicating that some functional tRNAs are imported from the cytoplasm for proper intra-mitochondrial translation. In addition, other genes related to the translation process, such as the ribosomal proteins *rps1*, *rps7*, *rps8*, *rps11* and *rpl6*, are absent, whereas *rps2*, *rps19* and *rpl16* appear to be pseudo or truncated genes.

**Table 2:** Main characteristics and features of *Utricularia reniformis* mtDNA genome.

*U*. *reniformis* Mitochondrial Genomic Features	Number	Length (bp)	% of MT Genome
A—common mitochondrial genes	70	61,207	7,14
- mtDNA coding regions	42	54,079	6.31
- group I intron	1	892	0,1
- group II intron (cis/trans-spliced)	19	17,543	2,04
- pseudogenes	3	711	0,08
- mtDNA tRNAs	25	1,929	0,23
-group I intron	2	671	0,07
- mtDNA rRNAs	3	5,219	0,61
B–ORFs (from 89bp to 665bp; average 192bp)	2,149	414,236	48.32
- no transcription evidence supported by RNAseq	944	156,725	18.29
- with more than 150pb (average 206bp)	484	101,237	11.81
- transcription evidence supported by RNAseq (≥ 5 reads)	1,205	257,471	30.03
- with more than 150bp (average 237bp)	935	223,352	26.05
C—mitovirus derived (RNA-dependent RNA polymerase)	7	3,800	0,44
D—chloroplast-derived regions (from *U*. *reniformis* plastome)	262	44,431	5,18
- identified cpDNA genes and truncated pseudogenes	71	28,586	3.33
- intact cpDNA genes	2	531	0.62
- pseudogenes/truncated/partial	69	28,055	3.28
E—repeated regions (excluding A, B, C and D)	883	49,000	5,72
F—Transposable Elements (TEs) related regions § (excluding A, B, C, D and E)	125	41,165	4.8
- Retroelements (Class I)			
-LINEs	6	1,550	0.18
-LTR elements	119	39,615	4.62
-Ty1/Copia	60	20,958	2.44
-Gypsy/DIRS1	36	12,954	1.51
**Summary**			
- Annotated and characterized regions, and potential hypothetical/chimeric orfs	3,496	613,839	71.61
- excluding potential hypothetical/chimeric orfs	1,351	201,147	23.46
- including potential hypothetical/chimeric orfs with transcription evidence	2,552	457,074	53.32
- Rest of the genome (intergenic spacer regions and non-characterized regions)	-	243,395	28.39
- Mapped by RNAseq (unique mapping– 99% identity and 100% coverage)	-	178,814	21

§ Only fragments of TEs were identified

**Table 3:** List of the traditional mitochondrial, chloroplast derived, and additional genes encoded by the *Utricularia reniformis* mtDNA genome.

**Genes of Mitochondrial Origin**	
Complex I (NADH dehydrogenase)	*nad1*•◊, *nad2*•◊, *nad3*, *nad4*•, *nad4L*, *nad5*•◊, *nad6*, *nad7*•, *nad9*
Complex II (succinate dehydrogenase)	*sdh3*, *sdh4*
Complex III (ubichinol cytochrome c reductase)	*cob*
Complex IV (cytochrome c oxydase)	*cox1*•, *cox2*, *cox3*
Complex V (ATP synthase)	*atp1*, *atp4*, *atp6*, *atp8*, *atp9*
Cytochrome c biogenesis	*ccmB*, *ccmC* (2x), *ccmFc*•, *ccmFn*
Ribosomal proteins (SSU)	*rps2* Ψ, *rps3*•, *rps4*, *rps10*•, *rps12*, *rps13*, *rps14*, *rps19* Ψ
Ribosomal proteins (LSU)	*rpl2* (2x), *rpl5*, *rpl10*, *rpl16* Ψ
Maturases	*matR*
Transport membrane proteins	*mttB*
Transfer RNAs	*trnC*-GCA, *trnD*-GTC, *trnE*-TTC, *trnF*-GAA, *trnG*-GCC, *trnH*-cp-GTG, *trnI*-CAT, *trnK*-TTT, *trnL*-CAA (2x), *trnfM*-CAT, *trnM*-CAT, *trnM*-cp-CAT, *trnP*-TGG, *trnP*-cp-TGG, *trnQ*-TTG, *trnR*-CCT•, *trnS*-GCT, *trnS*-TGA, *trnT*-GGT (2x), *trnT*-TGT, *trnV*-cp-TAC•, *trnW*-cp-CCA, *trnY*-GTA, *trnY*-GTA Ψ
Ribosomal RNAs	*rrn5*, *rrn5* Ψ, *rrnS*, *rrnL*
Others	LAGLIDADG endonuclease (intron region of *cox1* gene), Group II intron-encoded protein *ltrA*
**Genes of Chloroplast Origin**	
Photosystem I	*psaA* Ψ, *psaB* Ψ (2x), *psaC* Ψ
Protosystem II	*psbA* Ψ, *psbB* Ψ (2x), *psbC* Ψ (2x), *psbD* Ψ, *psbE* Ψ
Cytochrome b/f complex	*petD* Ψ, *petN* Ψ
ATP synthase	*atpA* Ψ, *atpB* Ψ (2x), *atpE* Ψ (2x), *atpF* Ψ
NADH dehydrogenase	*ndhA* Ψ (2x), *ndhB* Ψ, *ndhC* Ψ, *ndhD* Ψ, *ndhG* Ψ, *ndhH* Ψ (2x), *ndhJ* Ψ (2x), *ndhK* Ψ, *ndhN* Ψ
RubisCO large subunit	*rbcL* Ψ
RNA polymerase	*rpoA* Ψ, *rpoB* Ψ (4x), *rpoC1* Ψ, *rpoC2* Ψ (4x)
Ribosomal proteins (SSU)	*rps4* Ψ, *rps8* Ψ, *rps11*, *rps12* Ψ, *rps19* Ψ
Ribosomal proteins (LSU)	*rpl2* Ψ (2x), *rpl14* Ψ, *rpl23* Ψ, *rpl36*
Other genes	*accD* Ψ, *clpP* Ψ (2x), *infA* Ψ, *matK* Ψ (2x)
hypothetical chloroplast reading frames	*ycf2* Ψ (2x), *ycf15* Ψ, *orf56* Ψ
Ribosomal RNAs	*rrna16* Ψ, *rrn23* Ψ (4x)
Transfer RNAs	*trnL*-cp-TAA Ψ (2x), *trnK*-cp-TTT Ψ, *trnS*-cp-GCT Ψ, *trnE*-cp-TTC Ψ
**Additional Pseudogenes with Assigned Function**	Mitovirus RNA-dependent RNA polymerase Ψ (7x), DNA polymerase type B, organellar and viral Ψ (2x)
	DNA-directed RNA polymerase subunit beta Ψ (2x), DNA-dependent RNA polymerase

Ψ pseudogene

◊ trans-splicing

• intron-containing gene

Nineteen group II introns were found across the mtDNA sequence (Table 2), with one of them being of particular interest because it contains an ORF encoding a reverse transcriptase domain-containing protein (IPR000477 and cd01651 RT_G2_intron) similar to that of the *ltrA* gene. *ltrA* is a multifunctional protein that promotes splicing and mobility that was originally identified in *Lactococcus lactis* [55]. The reverse transcriptase domain of the *ltrA* gene is 49%, 50% and 51% similar to the 18S ribosomal RNA intron1, *atpA* intron1, and *cob* intron3, respectively, of the white spruce (*Picea glauca*) mitochondrial genome, indicating their putative role for the splicing of these genes. We also found seven trans-spliced genes, including *nad1*. Interestingly, the maturase-coding *matR* gene is located between exon1 and exon4 of *nad1*, and this syntenic feature is conserved in the *U*. *gibba* mtDNA.

Group I introns have also been identified in *Utricularia reniformis* mtDNA genes. The software ARAGORN detected that the 193bp-long *trnR*-CCT gene contains a group I intron located in the anticodon loop, thus most likely not interrupting the overall tRNA structure. Although the tRNA splicing machinery and mechanism in plant cells are currently unclear [56], splicing would be indispensable for the maturation of this tRNA. Interestingly, the group I intron found in the *cox1* gene was identified encoding a LAGLIDADG endonuclease. It is noteworthy that the draft mtDNA of *U*. *gibba* Scaffold00369 (KC997779) presents an identical *cox1* organization containing exon1, LAGLIDADG, and exon2, with 97%, 97% and 100% identical amino acid residues, respectively, when compared to the *U*. *reniformis* sequences, which supports intron acquisition from a common ancestor. Moreover, the *cox1* gene of both mtDNAs presented eleven, out of twelve, of the putative positively selected motifs (Phe/Lys-164 motif is absent), in which accumulation of nucleotide substitutions, including the most important motif Cys-113-Cys-114, have been previously reported [18].

### *Utricularia reniformis* mtDNA features

#### Repeats and similarities to *U*. *gibba* mtDNA

**Fig 2:**
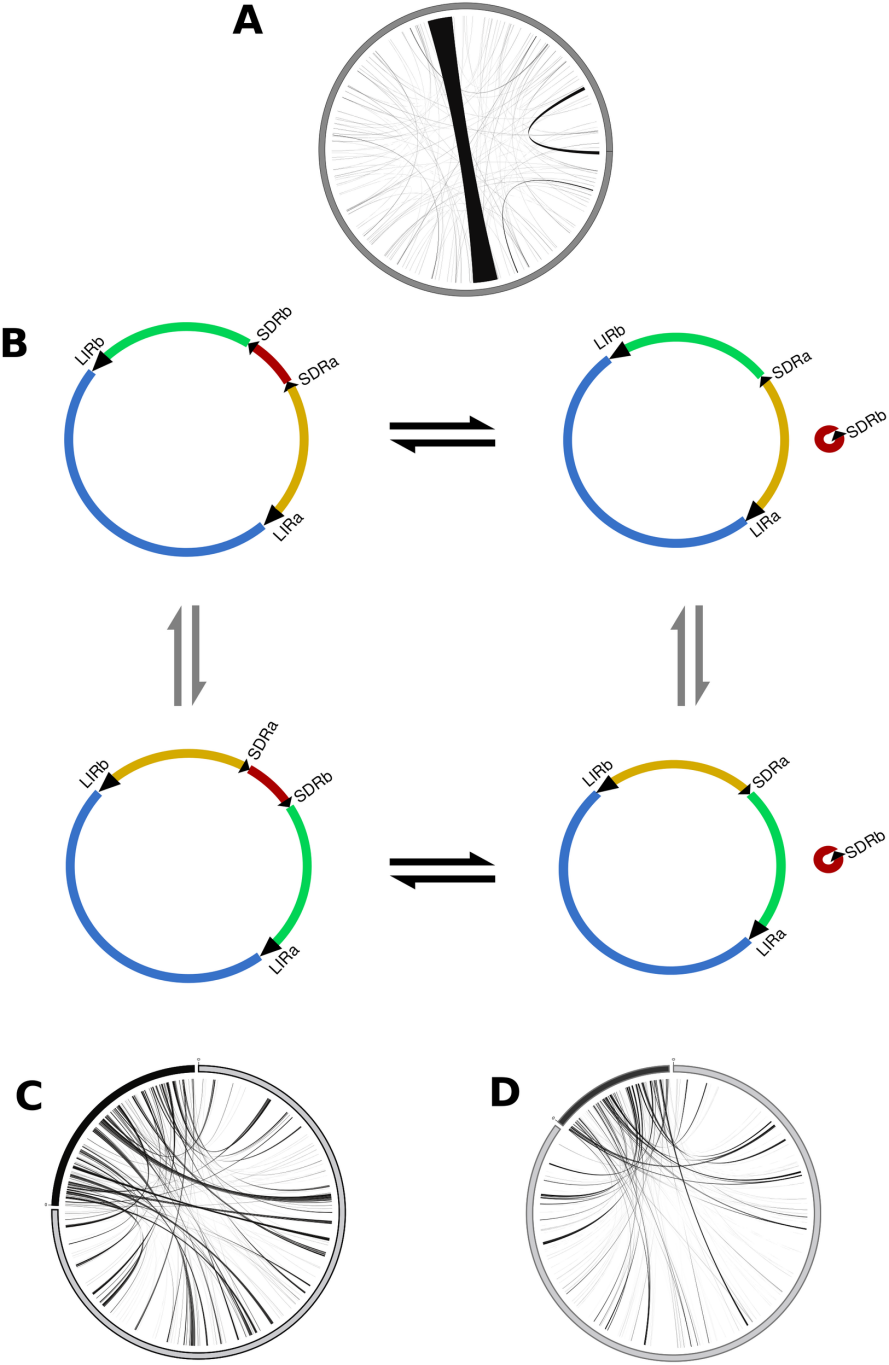
Repeats and alternative master circle structures. (A) Repeats across *U*. *reniformis* mtDNA; (B) Putative alternative conformations of *U*. *reniformis* mtDNA based on repeat-mediated intramolecular recombination mechanism; (C) Shared regions among *U*. *reniformis* mtDNA and *U*. *gibba* mtDNA; (D) repeats between *U*. *reniformis* cpDNA against the mtDNA.

A total of 883 regions, ranging from 37bp to 25,125bp and corresponding to 49kbp, are repeated across *U*. *reniformis* mtDNA (Table 2 and Fig 2A). In addition to a large inverted repeat (LIR) and a small direct repeat (SDR) regions, at least seven additional repeated regions span more than 100 bp. These repeated regions could be involved with repeat-mediated homologous recombination that can generate sub-genomic circles or other alternative conformations. For instance, putative intramolecular recombination between the LIRa and LIRb and SDRa and SDRb repeats could generate at least four alternative MC conformations, including a small 70 kbp sub-circle, which may be involved in a putative direct-repeat-mediated deletion of a region containing the *rps12*, *rps14*, *rpl5*, *nad3* and *trnQ-TTG* genes (Fig 2B). According to a dot plot and AMOS software package [57] analysis, the *U*. *gibba* mtDNA sequence (downloaded from the CoGe OrganismView database; https://genomevolution.org/coge/GenomeInfo.pl?gid=29027-unitig_87) can be circularized into a single molecule of 270,037 bp. Although most of the *U*. *reniformis* traditional mitochondrial genes show high level of identity (~ 93.95%) with their homologues in *U*. *gibba*, these genomes exhibit highly repetitive content and essentially no conservation in synteny (Fig 2C), indicating that *U*. *gibba* has a significantly different mtDNA and that divergent evolutionary forces are acting in the intronic and non-traditional mitochondrial coding regions of both genomes.

#### Open reading frames

The largest portion of the *Utricularia reniformis* mtDNA (414kbp or 48%) is composed of 2,149 ORFs ranging from 89bp to 665bp (average 192bp) (Table 2). Blast searches identified that 1,898 of these have no similarity to any sequence from the Viridiplantae in the NCBI database, and 152 had hits to hypothetical proteins in other plant genomes, mostly exhibiting resemblance to partial sequences of organellar genes, such as the DNA and RNA polymerases, likely derived from mitochondrial plasmids [23], retrotransposons or nuclear genes (Table 4). Interestingly, a large number of unknown, hypothetical ORFs with putative function are transcribed, whereas some exhibit signal peptide and transmembrane domains (Table 4 and in more details in S2 Table), suggesting that these putative proteins may be exported to participate in cell signaling or inserted into the mitochondrial membranes. Therefore, several ORFs should produce peptides, whereas others are may be recombination remnants.

**Table 4:** Main characteristics of the unknown (no hits) and hypothetical open reading frames identified in *U*. *reniformis* mtDNA.

ORFs	Number	Signal Peptide + Transmembrane Domain	Only Signal Peptide	Only Transmembrane Domain
**Unknown (no hits)**	1,898	21	187	254
- transcribed	1,018	17	98	135
- non transcribed	880	4	89	119
**Hypothetical**	152	2	8	15
- transcribed	111	1	8	14
- non transcribed	41	1	0	1
**Putative Function**	99	0	4	9
- transcribed	76	0	3	6
- non transcribed	23	0	1	3

#### Sequences of plastid origin

**Fig 3:**
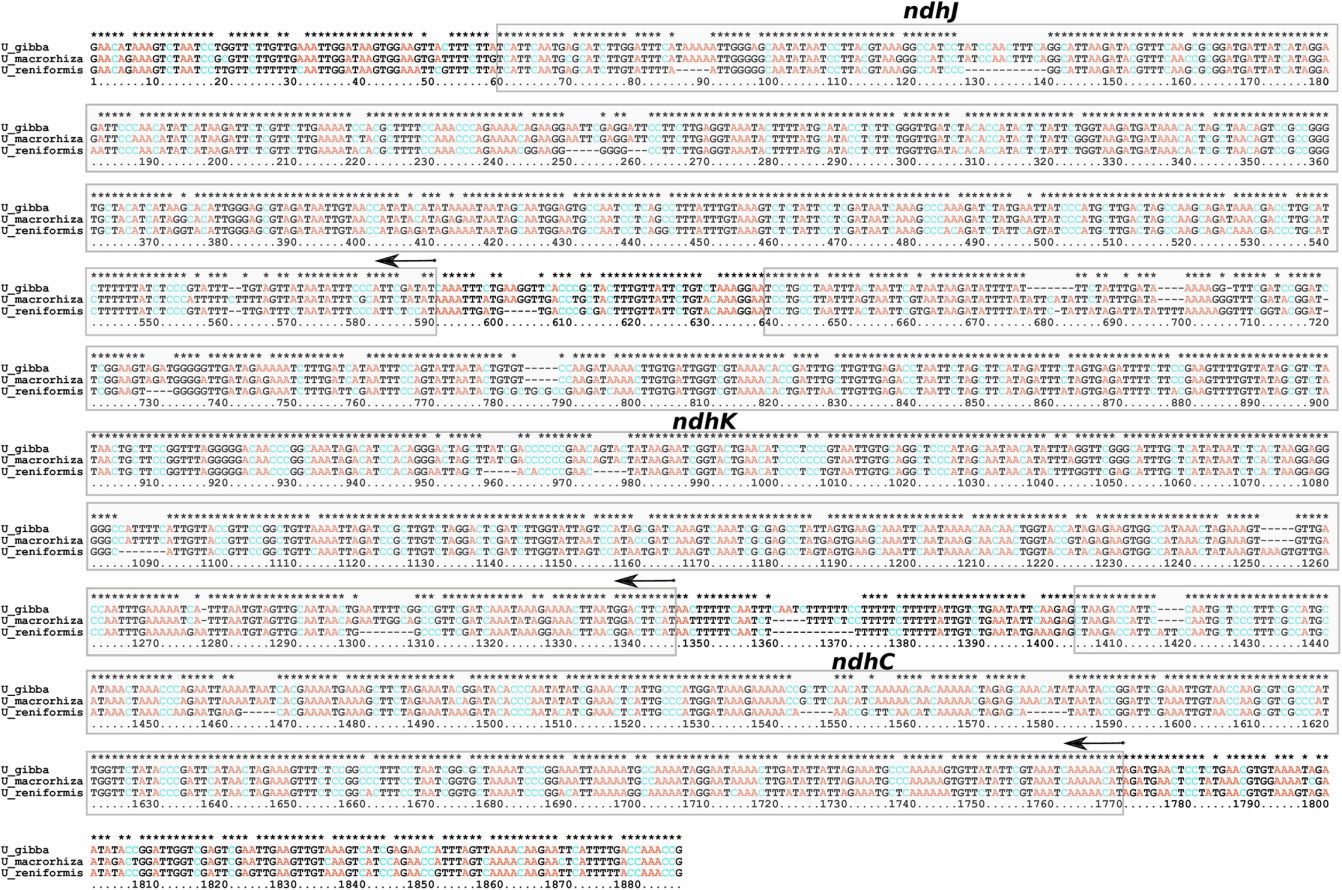
*U*. *reniformis* mtDNA *ndhJ*-*ndhK*-*ndhC loci* alignment against the respective loci in the cpDNA of *U*. *gibba* and *U*. *macrorhiza*.

Integrated plastid sequences generally correspond to from 1% to up to 12% of an angiosperm mtDNA, and are indeed widespread in seed plants [23,58]. Plastid-like sequences were located in intergenic regions corresponding to a least 44kbp (5%) of *Utricularia reniformis* mtDNA (Table 2 and Fig 2D), corroborating our previous observations of the occurrence of extensive lateral gene transfer between the organelles [16]. These insertions are spread in fragments of up to 1.5kbp and represent 31% of the *U*. *reniformis* plastid genome. In addition to numerous intergenic spacer regions, two intact plastid genes, *rps11* and *rpl36*, and 69 truncated pseudogenes and tRNAs were identified (Table 3). We have previously reported that the plastid NAD(P)H-dehydrogenase complex *ndhJ*-*ndhK*-*ndhC* gene locus, which is absent in the *U*. *reniformis* plastid genome, is present as truncated pseudogenes in the mtDNA [16]. Indeed, the *U*. *reniformis* mtDNA *ndhJ*-*ndhK*-*ndhC* locus shows ~87% of nucleotide similarity to the homologous region in *U*. *gibba* and *U*. *macrorhiza* cpDNA (Fig 3), although these genes in *U*. *reniformis* mtDNA should not be functional.

#### Transposable elements

The *U*. *reniformis* mtDNA contains 125 fragments of transposable element-related sequences from different families, accounting for up to 41kbp (4.8%) of the genome (Table 2), the majority of which being Ty1/Copia (21kbp) and Gypsy/DIRS1 (13kbp). Most are located in intergenic regions, and no complete elements were identified, indicating that these sequences represent relics of ancient events of lateral transfer from the nucleus. Using the same methodology to track TE fragments, the circular mtDNA of *U*. *gibba* exhibited a TE load of 10,452bp (3.9%), with Ty1/Copia (6.5kbp) and Gypsy/DIRs (3.1kbp) the major representatives. Indeed, the presence of relics of TEs is often observed in angiosperm and eudicot mtDNA [23], suggesting a putative role for these elements in shaping the mitochondrial genome structure and evolution.

#### Mitovirus derived sequences

At least 3.8kbp (0.4%) of the mtDNA correspond to up to 7 regions related to partial mitoviruses sequences. Interestingly, more than 3 unique full-length RNAseq reads were mapped in each region (S3 Table), suggesting that these truncated mitovirus regions are still transcribed, but whether they encode regulatory or functional proteins is currently unclear. The mitovirus sequences belong to the Narnaviridae family and are the simplest, unencapsidated viruses, ranging from 2.3 to 3.6 kbp and encoding only a single RNA dependent RNA polymerase protein (RdRp) [59,60]. The Narnaviridae family is widespread among filamentous fungi, in particular phytopathogenic fungi [60]. Therefore, it is believed that the mitoviruses originated from horizontal gene transfer (HGT) from plant pathogenic fungi [59]. The RdRp regions identified in *U*. *reniformis* mtDNA share identity to the PFAM (http://pfam.xfam.org) family PF05919, present in several mtDNA of species from the Viridiplantae, including *Arabidopsis thaliana*, which contains complete mitovirus copies in both nuclear and mitochondrial genomes. To date, we are unable to determine whether complete mitovirus sequences are present in *U*. *reniformis* nuclear genome assembly. Interestingly, in *A*. *thaliana*, only the mitochondrial copy is expressed [59].

### Mitochondrial-related genes are transcribed together with newly identified ORFs and inter-genic regions

**Fig 4:**
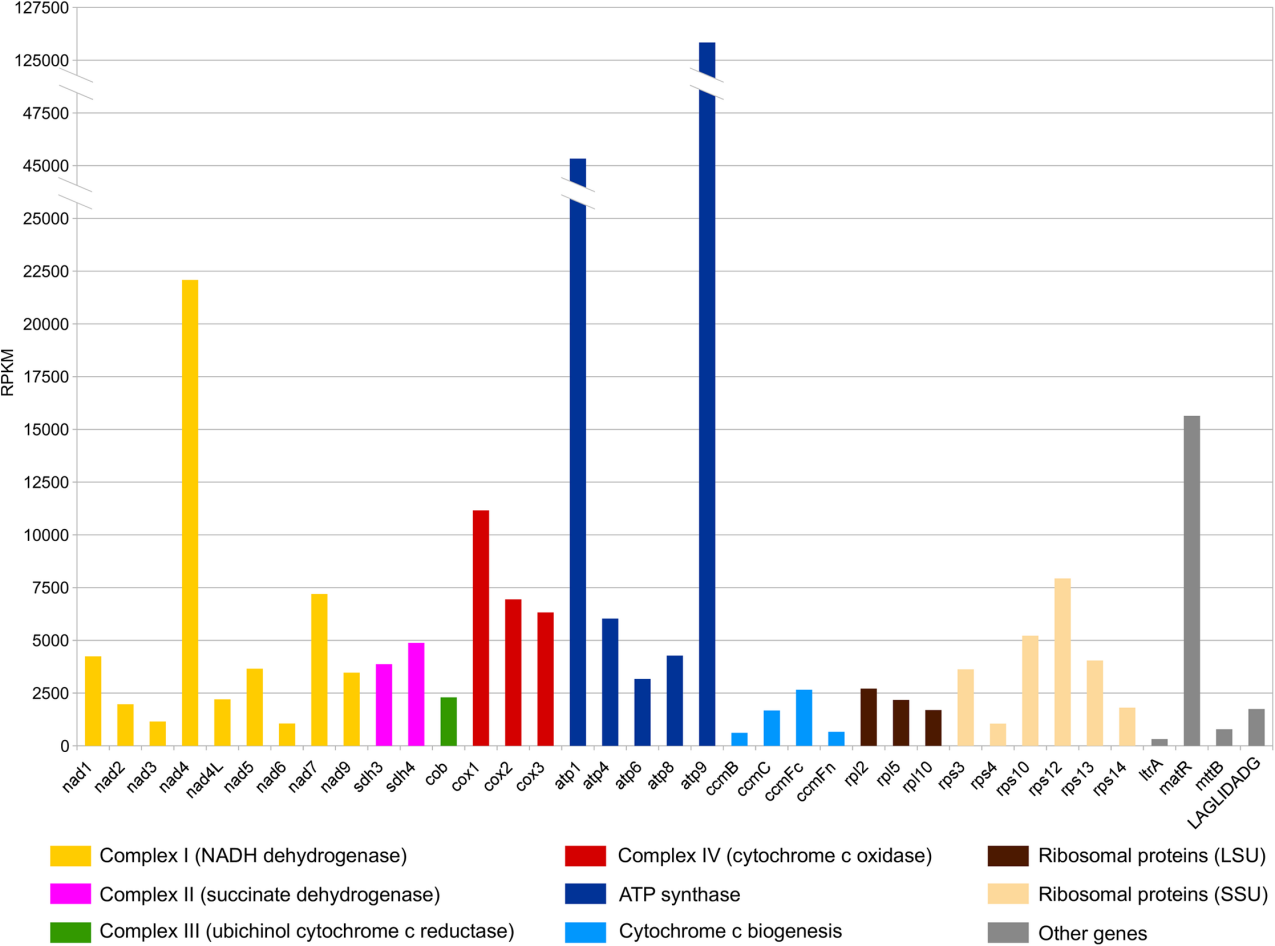
Transcription patterns of the traditional mitochondrial genes by Reads Per Kilobase Million (RPKM).

A total of 1,2Mbp of RNA-seq reads were mapped to the *Utricularia reniformis* mtDNA, confirming that all traditional mitochondrial genes are transcriptionally active. Additionally, 78% (672kbp) of the mtDNA is covered by at least one RNA-seq read, indicating that the newly identified ORFs and portions of the intergenic spacer regions are also transcribed (Table 1). It is noteworthy that from 243kbp of the intergenic spacer regions, a total of 178kbp have at least one RNA-seq read mapped (Table 2), indicating unexpected transcription of a large portion of the mtDNA, or that genome-length poly-cystronic transcripts are produced, as is the case for animal mtDNA [61]. Fig 1 (inner red circle) shows the RNA-seq read mapping depth of *U*. *reniformis* mtDNA, indicating, as expected, that *rrnS* and *rrnL* are the sites for most transcriptional activity. However, an additional peak located near a mitovirus related region at genomic position 531kbp (Fig 1; eight forth five o’clock) is associated with a partial retrotransposon derived region. This finding suggests that relics of retrotransposons are still transcribed. However, it is unlikely that this fragment was actively transposed, since it carries only a partial sequence of a retrotransposon without terminal repeats. The transcription pattern of this region and all other TE-related should be interpreted with caution due to the difficulty in determining the source of this transcript (nuclear genomic retroelement or real organellar). The transcription pattern of all traditional mitochondrial genes is shown in Fig 4, showing that *atp1* and *atp9* are the most expressed genes, and that *ccmB*, *ccmFn* and *ltrA* are the least expressed ones. Even the truncated pseudogenes *rps19* Ψ, *rpl16* Ψ and *rps2* Ψ present unique mapped RNAseq reads, indicating that they are transcribed (≥ 98% identity considering 100% of the read length) (S3 Table). This appears to be a common feature of *U*. *reniformis* organelles, since the transcription evidence of truncated *ndh* pseudogenes were also observed in the *U*. *reniformis* cpDNA [16].

### Mitochondrial RNA editing analysis revealed 69 novel sites

The PREP-Mt tool and the mapped RNA-seq data identified, respectively, at least 385 and 147 RNA editing sites, 69 of which corresponding to novel editing sites in traditional protein-coding genes of the *U*. *reniformis* mtDNA (Table 5). *nad1*, *nad4* and *nad7* were the most edited genes, indicating a common process for mitochondrial complex I genes. Interestingly, CGA (R) to UGA editing was detected in exon2 of the *rps*3 gene in about 85% of the mapped RNA-seq reads. This change causes a premature stop codon, located 1,220 nucleotides upstream of the predicted stop codon, thus interrupting at least 73% of *rps3* exon2. This finding suggests a balanced production of a long and a short isoform for the *rps3*-encoded protein. Moreover, six editing sites were identified in the *cox1* gene, two of which cause non-synonymous substitutions at the positively selected amino acid motifs Ser/Phe-78 and Pro/Leu-194 [18] at the rate of 91% for S→F (position 321,375) and 13% for the P→L (position 321,723).

**Table 5:** The 69 novel RNA editing sites identified in the protein-coding genes of *U*. *reniformis* mtDNA. For a complete list of the 147 editing sites identified by the RNAseq approach please refer to S4 Table.

Gene	mtDNA		Codon		Codon position	Amino acid		Editing level (%)
	Position	Strand	From	To		From	To	
*nad1* (4 sites)	99048	+ (exon 1)	CUA	UUA	1	L	L	18
	99201	+ (exon 1)	CCG	UCG	1	P	S	88
	344481	+ (exon 3)	CCC	CCU	3	P	P	33
	726995	- (exon 5)	AAC	AAU	3	F	N	63
*nad2* (5 sites)	297295	- (exon 2)	CCA	CUA	2	P	L	92
	463531	+ (exon 4)	UUC	UUU	3	F	F	18
	463653	+ (exon 4)	CCG	CUG	2	P	L	81
	465022	+ (exon 5)	UCC	UUC	2	S	F	70
	465030	+ (exon 5)	CUC	UUC	1	L	F	64
*nad3* (1 site)	20810	-	CCA	CUA	2	P	L	92
*nad4* (3 sites)	578975	+ (exon 3)	CUA	UUA	1	L	L	36
	579324	+ (exon 3)	CCA	CUA	2	P	L	94
	581798	+ (exon 4)	ACC	ACU	3	T	T	93
*nad5* (5 sites)	260779	- (exon 2)	GCC	GCU	3	A	A	29
	260950	- (exon 2)	AUC	AUU	3	I	I	20
	261311	- (exon 2)	GCC	GUC	2	A	V	75
	261673	- (exon 2)	ACC	ACU	3	T	T	25
	261677	- (exon 2)	CCG	CUG	2	P	L	92
*nad6* (2 sites)	300266	-	CCA	CUA	2	P	L	93
	300273	-	CCC	UCC	1	P	S	90
*nad7* (13 sites)	711488	+ (exon 1)	AAC	AAU	3	N	N	13
	712855	+ (exon 2)	UCU	UUU	2	S	F	70
	713691	+ (exon 3)	CGU	UGU	1	R	C	100
	713819	+ (exon 3)	GUC	GUU	3	V	V	24
	713906	+ (exon 3)	UCC	UCU	3	S	S	12
	714114	+ (exon 3)	CUU	UUU	1	L	F	91
	715347	+ (exon 4)	CCU	CUU	2	P	L	72
	715366	+ (exon 4)	UCC	UCU	3	S	S	61
	715453	+ (exon 4)	CCC	CCU	3	P	P	88
	715506	+ (exon 4)	UCU	UUU	2	S	F	85
	715522	+ (exon 4)	CAC	CAU	3	H	H	72
	715527	+ (exon 4)	CCA	CUA	2	P	L	98
	715569	+ (exon 4)	UCU	UUU	2	S	F	100
*nad9* (3 sites)	497201	+	UCU	UUU	2	S	F	73
	497282	+	CCA	CUA	2	P	L	47
	497708	+	UCU	UUU	2	S	F	78
*cob* (2 sites)	779528	+	UCU	UUU	2	S	F	92
	779794	+	CUA	UUA	1	L	L	86
*cox1* (2 sites)	321763	+ (exon 1)	ACC	ACU	3	T	T	89
	323181	+ (exon 2)	CUG	UUG	1	L	L	96
*cox2* (1 site)	514300	+	CUA	UUA	1	L	L	84
*cox3* (1 site)	558125	+	UUC	UUU	3	F	F	67
*atp4* (2 sites)	285091	+	CCC	CUC	2	P	L	87
	285114	+	CCG	UCG	1	P	S	95
*atp6* (2 sites)	810361	-	UCC	UCU	3	S	S	48
	810993	-	CGU	UGU	1	R	C	64
*atp8* (1 site)	556640	+	UGC	UGU	3	C	C	84
*ccmFc* (3 sites)	834747	+ (exon 1)	CAU	UAU	1	H	Y	63
	834901	+ (exon 1)	CCA	CUA	2	P	L	77
	836320	+ (exon 2)	CUA	UUA	1	L	L	32
*ccmFn* (1 site)	488236	-	ACC	AUC	2	T	I	61
*rpl5* (4 sites)	22103	-	CCG	UCG	1	P	S	16
	22105	-	CCU	CUU	2	P	L	77
	22173	-	AUC	AUU	3	I	I	13
	22389	-	UCC	UCU	3	S	S	31
*rpl10* (3 sites)	611859	+	ACC	ACU	3	T	T	35
	611934	+	CGG	UGG	1	R	W	41
	612084	+	UAC	UAU	3	Y	Y	40
*matR* (7 sites)	102277	+	UCC	UCU	3	S	S	96
	103757	+	CGC	UCG	1	R	S	16
	103771	+	UAC	UAU	3	Y	Y	75
*rps3* (4 sites)	263919	- (exon 2)	CCG	CUG	2	P	L	97
	263864	- (exon 2)	UCC	UCU	3	S	S	48
	264252	- (exon 2)	CCA	CUA	2	P	L	15
	264811	- (exon 2)	CGA	UGA	1	R	Stop	85
*rps4* (1 site)	267470	-	UCC	UCU	3	S	S	56
*rps12* (1 site)	20376	-	CCC	CCU	3	P	P	11
*rps14* (2 sites)	21929	-	UCC	UCU	3	S	S	21
	21974	-	CCC	CCU	3	P	P	17

### Phylogenetics analysis based on mtDNA supports Lentibulariaceae as a monophyletic group

**Fig 5:**
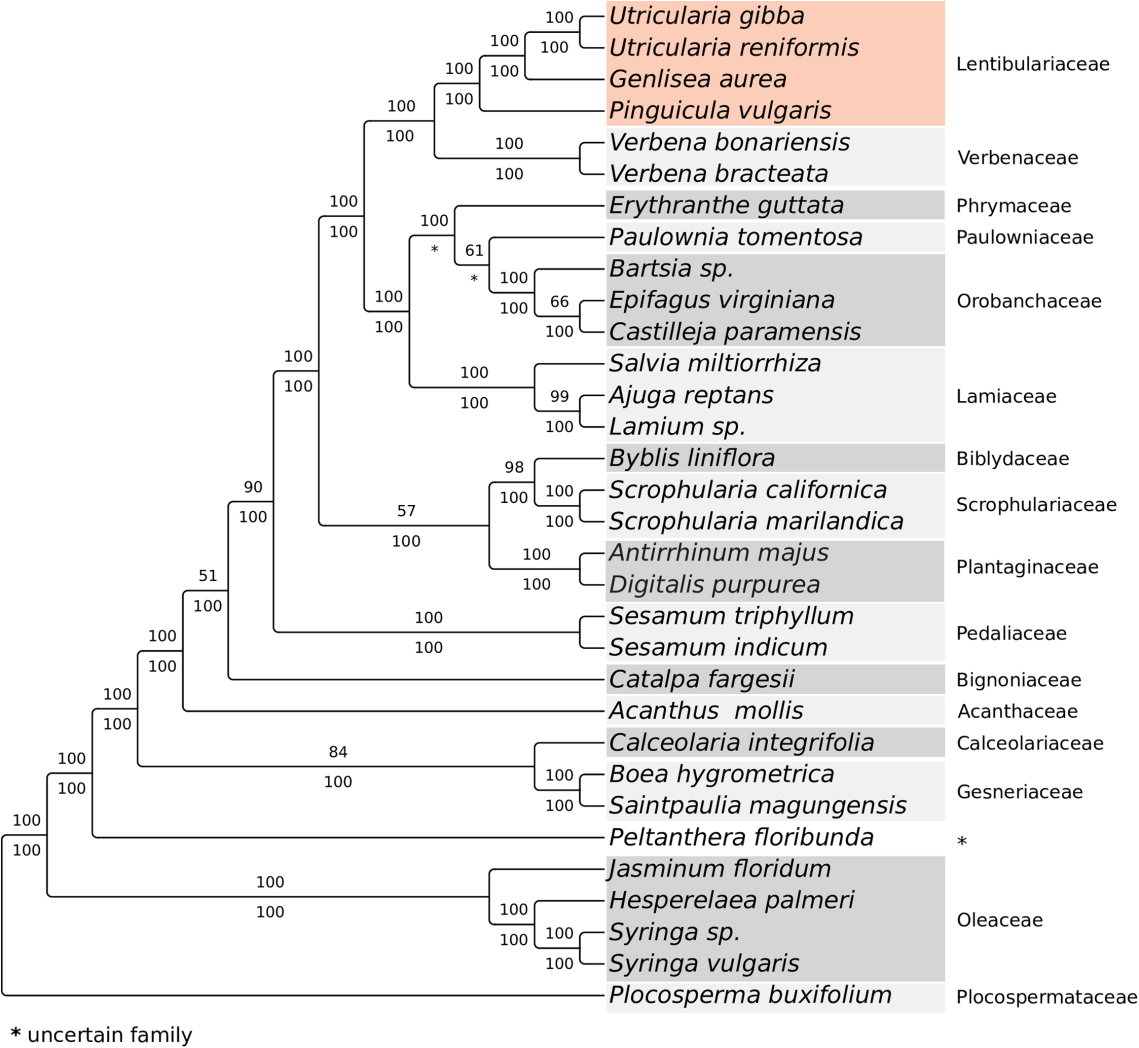
Phylogenetic analysis based on the mitochondrial genes *atp1*, *cox1*, *matR*, *nad5*, *rps3* from 32 different species from the Lamiales order.

The use of mitochondrial genes for phylogenetic purposes has been broadly discussed as these genes are more conserved than genes found in chloroplast genomes, and despite the fact that mitochondrial genome complexity could lead to biased tree reconstruction at interspecific level [62], many deeper clades in plant phylogeny are accepted because of mitochondrial gene information [63]. However, it is well known that the phylogeny of plant mitochondria may not accurately represent the organism’s evolutionary history, mainly due to the frequent horizontal transfer of mitochondrial genes [64] (Fig 5).

As expected, our mitochondrial phylogenetic analysis shows that the family Lentibulariaceae is monophyletic and that *U*. *reniformis* is placed with *U*. *gibba* as sister group of *Genlisea aurea*, nested with *Pinguicula vulgaris* (Fig 5), as previous published phylogenies have shown [65,66]. Although the relationships among families of Lamiales are rather controversial (see [67]), the relations found in this study are consistent with the APG IV classification [66], except for the relations of Bignoniaceae, Pedaliaceae, Plantaginaceae, Scrophulariaceae and Biblydaceae. Missampling of taxa and the probable horizontal transfer of mitochondrial genes between divergent species could account for these discrepancies. Further studies are required to clarify the relations among these families.

## Discussion

### The mtDNA master circle paradigm and multipartite structures in *U*. *reniformis*

Current methods and protocols to isolate and sequence plastid or mitochondrial DNAs are still laborious. To avoid these technical issues, whole-genome sequencing methods stand as one of the most reliable approaches to uncover plant organellar genomes. Using this approach, the nuclear and organellar reads are mixed, making it mandatory to filter for authentic organellar reads prior to genome assembly. Although this would be expected to be accomplished fairly easily, since the organellar reads are overrepresented in deep whole-genome sequencing data [68], the occurrence of similar and horizontally transferred sequences among the organelles and the nuclear genome makes this procedure rather demanding from a bioinformatics point-of-view due to the occurrence of similar and horizontally transferred sequences between the organelles and the nuclear genome [28,69,70]. Even with good organellar read filters, genome assembly in the case of plant mtDNA is not straightforward, due to its complex physical organization. Therefore, the genomic MC is actually considered a representative format in which plant mitochondrial genomes are reported and used for comparative analysis and to reconstruct phylogeny [25].

The approach developed in this study successfully allowed the reconstruction of the *U*. *reniformis* mtDNA MC. However, it is well known that circular structures are difficult to observe or do not even exist *in vivo* [23,71,72]. Indeed, several complex alternative linear and circular genomic conformations, such as subgenomic circles, branched structures, head-to-tail concatamers and circularly permuted linear molecules are often found in attempts to observe the *in vivo* structure of plant mtDNA [23,25,72]. These alternative conformations are generally produced by intramolecular recombination driven by repeats. The presence of large repeats is commonly observed in angiosperm mtDNA, including related asterid species [23,53] and *U*. *reniformis*. Interestingly, these features are apparently absent in the *U*. *gibba* mtDNA, whereas all traditional mitochondrial genes are encoded in a circular molecule of 270kbp. Therefore, the results presented here suggest that repeat-mediated recombination processes may play a role in generating diverse *U*. *reniformis* mtDNA forms that alternate from the MC, although further investigation is necessary to show the presence, types and frequencies of these putative alternative genome conformations.

### Possible TE expansion and whole genome duplication detected in *U*. *reniformis* nuclear genome

Angiosperm mtDNA normally range from 222kbp to 983kbp with GC content of 43–45% [23], and shares several features, such as size variation among different families, and even within species, presence of large amounts of unknown ORFs and non-coding DNA, repeated sequences, TEs, incorporation of cpDNA and nuclear sequences, and gain or loss of a number of chromosomes in mega-sized mitochondrial genomes [23,73]. Furthermore, trans-splicing, RNA editing, cytoplasmic male sterility (CMS) related genes, and partial copies of RdRp genes derived from uncapsidated mitoviruses are other common features often present in angiosperm mtDNA [23,59]. *Utricularia reniformis* mtDNA exhibits almost all of these characteristics, but when compared to *U*. *gibba*, genomic size stands out. Interestingly, *U*. *reniformis* and *U*. *gibba* mitochondrial genomes are 857 and 270kbp long, respectively, and both encode almost all traditional mitochondrial genes. An accumulation of TE fragments in both *Utricularia* mtDNA provides valuable insights into this size difference, suggesting a distinct evolutionary trajectory for both species. It is well established that retrotransposons and nuclear genes present in the mtDNA are derived from lateral transfer events between the cellular compartments [53,64], so the abundance of TEs in the *Utricularia* mtDNA may be a reflection of what is happening in the nucleus. In fact, the nuclear genome of *U*. *gibba* is 101Mbp in size, of which at least 8.9% corresponds to complete TEs [14], whereas *U*. *reniformis* exhibits a ~316Mbp-long nuclear genome. Previous studies support that *U*. *reniformis* may have a tetraploid nuclear genome with high levels of heterozygosity [17], corroborating our k-mer spectrum plot analysis that strongly suggests whole-genome duplication (WGD) and/or polyplodization events in ~316Mbp-long and highly heterozygous genome. Therefore, we propose that TE propagation and extinction might be one of the possible mechanisms to explain genome expansion and contraction observed in both *Utricularia* species. Future work on *U*. *reniformis* nuclear genome and comparative analysis against *U*. *gibba* will definitely help us test this hypothesis.

### The role of the *cox1* intron in *U*. *reniformis*

The *cox1* intron has been frequently acquired via horizontal transfer in angiosperms, whereas previous authors proposed that the *cox1* intron was originally acquired from fungi and laterally transferred several times during angiosperm evolution [74,75]. The intron-containing *cox1* gene is typically formed by two exons spanning 726bp and 858bp [70]; in *Utricularia reniformis*, exon1 is 721bp and exon2 is 864bp-long. The presence of an intact ORF for a LAGLIDADG endonuclease located in this intronic region, and its expression as detected by the RNA-seq data, are puzzling, since it was proposed that LAGLIDADG occurrence is associated with very low substitution rates in plant mtDNA [70]. However, previous studies indicate that *Utricularia* has significantly high rates of nucleotide substitutions in all three genome-bearing cellular compartments [5], possibly due to the increased respiratory rates associated with positive selection at the *cox1* locus [18,19]. We therefore speculate that the acquisition of a LAGLIDADG-coding intron in *Utricularia* mtDNA represents a recent event as previously proposed [70], with possible implications for the genome function and evolution, although the putative role of this endonuclease in keeping a low genomic substitution rate remains uncertain.

Several other group II introns were identified; one in particular encodes an *ltrA*-like protein, which may be related to splicing and mobility. The *ltrA* gene has not been previously described as a common feature of angiosperms mtDNA [23], even though relics of truncated copies of the *ltrA*-like gene are found in several other species, but not in the *U*. *gibba* mtDNA. These relics were not previously annotated in most of these mtDNAs, suggesting that *ltrA* is in fact an unnoticed common feature propagated during the course of the angiosperms evolution. Nonetheless, because *ltrA* is one of the least expressed genes, its putative mobility and splicing functions and role in the *U*. *reniformis* mtDNA evolution remain to be thoroughly investigated.

### Large number of unknown ORFs and their putative roles in cytoplasmic male sterility

We identified at least 2,149 unknown ORFs, a large number of which exhibited transcripts and predicted proteins with signal peptides and/or transmembrane domains, suggesting important functional roles. It has been proposed that recombination events can give rise to novel ORFs that are often a combination of common mitochondrial genes and unknown ORFs, and that this phenomenon can be associated with the cytoplasmic male sterility (CMS), long reported for plant species [23]. The floral morphological aspects of *U*. *reniformis* suggest low reproductive success from cross-pollination in natural conditions [76], and a putative role for CMS is implied. Despite the nature and function of the CMS associated genes being poorly understood, it was previously cogitated that a malfunctioning ATPase or absence of *nad7* may be related [77]. However, the *nad7* gene is present in *U*. *reniformis* mtDNA, and truncated *atp*-related genes were not identified in association with unknown ORFs. We believe that further characterization of these ORFs and their putative products is warranted and will help establish the CMS genetic architecture in angiosperms.

### Lateral gene transfer and potential functional replacement of plastid genes to the mtDNA

We also identified several pseudogenes of plastid origin in *Utricularia reniformis* mtDNA, a feature that is conserved in *U*. *gibba* [15]. Notably, the presence of the *ndhJ*-*ndhK*-*ndhC* locus, which is absent in the cpDNA, indicates a lateral transfer to the mtDNA, followed by a decay of the original copies in the cpDNA. Similar translocation of *ndh* genes from the cpDNA to the mtDNA was also observed in the Orchidaceae family, in particular in *Erycina pusilla* [78], suggesting that this type of event is more common than previously thought.

Moreover, the cpDNA *rps11* and *rpl36* genes appear complete, indicating that they may still be functional. However, the transcriptome profile of the plastid-like insertions was not inferred due to it being extremely difficult to determine the source of the transcripts (plastid, mitochondrial, or even nuclear). Previous studies indicated that the integrated plastid regions originate novel mitochondrial genes involved in maturation of mitochondrial mRNAs, and are therefore unrelated to their original plastidic functions [58]. The high number of truncated plastid genes may support this trend in *U*. *reniformis*, but their functional and evolutionary roles remain to be established.

### Unexpected transcripts and RNA editing as important players in *U*. *reniformis* mtDNA evolution

The evidence of RNA transcripts for intergenic regions, newly identified ORFs, and partial fragments of retrotransposons in the mtDNA of *Utricularia reniformis* indicates an enrichment of unexpected genomic expression. Similar findings were also observed in other plant mtDNA, such as *Oryza sativa* and *Nicotiana tabacum* [79,80], supporting a common trend, and indicating that in-depth transcriptome analysis sheds additional light onto the mechanisms of mitochondrial genomic function and evolution. This analysis also revealed up to 532 RNA editing sites, a number consistent with the estimated number (roughly 500) for angiosperm organellar genomes [81]. Among these, 69 correspond to novel editing sites, including the site that leads to a premature stop codon in the *rps3* gene. RNA editing generating stop codons are also observed in the *atp9* and *rps10* genes of the *Rhazya stricta*, *Citrullus lanatus*, and *Cucurbita pepo* [31,53]. The fact that PREP-Mt detected more sites than the RNA-seq based approach may be related to the transcriptome conditions and tissues used, and to the high Phred quality values considered here to ensure data confidence. The RNA editing mechanism may be responsible for creating considerable polypeptide diversity in plant organelles, especially mitochondria, in which the genome shows such high structural plasticity [81]. We thus agree with previous work [82] that proposes the use of combined next-generation-sequencing approaches to unravel plant mitochondrial genomes and transcriptomes.

## Conclusion

In this study, we characterized the first carnivorous plant and eighth largest mtDNA from the Brazilian endemic and terrestrial carnivorous *Utricularia reniformis*, providing several insights into the genomics trends and evolutionary characteristics and trajectory of the family Lentibulariaceae.

## Supporting information

S1 Fig Assembly graph of *Utricularia reniformis* mtDNA based on the paired-read (2x300bp) assembly, generated by the Bandage software. The assembled contigs (nodes, represented as colored bars) with multiple inputs and outputs, and dead ends; and the connections between those contigs (edges, represented as black connectors) are shown.
(TIF)

S2 Fig A paired-end (mate-pairs) mapping read-track displaying the reads and coverage generated by CLC Genomics Workbench v9.5.2 tool. Blue lines represent the paired reads located on the border of the repeat region; the yellow lines represent the paired reads located on each repeated region. Mismatches between the reads and reference are shown as narrow vertical traits. The read coverage are shown as peaks located in the bottom of each figure, whereas blue represent the repeat borders and yellow the repeat itself.
(TIF)

S1 Table Mitochondrial genes (*atp1*, *cox1*, *matR*, *nad5*, *rps3*) from 32 different species from the Lamiales order used in the phylogenetic analysis. (XLSX)

S2 Table List of all identified open reading frames and detected signal peptide and transmembrane domains. (XLSX)

S3 Table RNAseq analysis of all identified genes of *U*. *reniformis* mtDNA. (XLSX)

S4 Table The 147 RNA edit sites identified on the traditional mitochondrial coding-regions by RNAseq read mapping. (XLSX)
