# Supplementary figures and images for: The mitochondrial genome of the terrestrial carnivorous plant Utricularia reniformis (Lentibulariaceae): Structure, comparative analysis and evolutionary landmarks
Source: PLoS One. 2017 Jul 19;12(7):e0180484. doi: 10.1371/journal.pone.0180484 (PMC5516982; doi:10.1371/journal.pone.0180484)

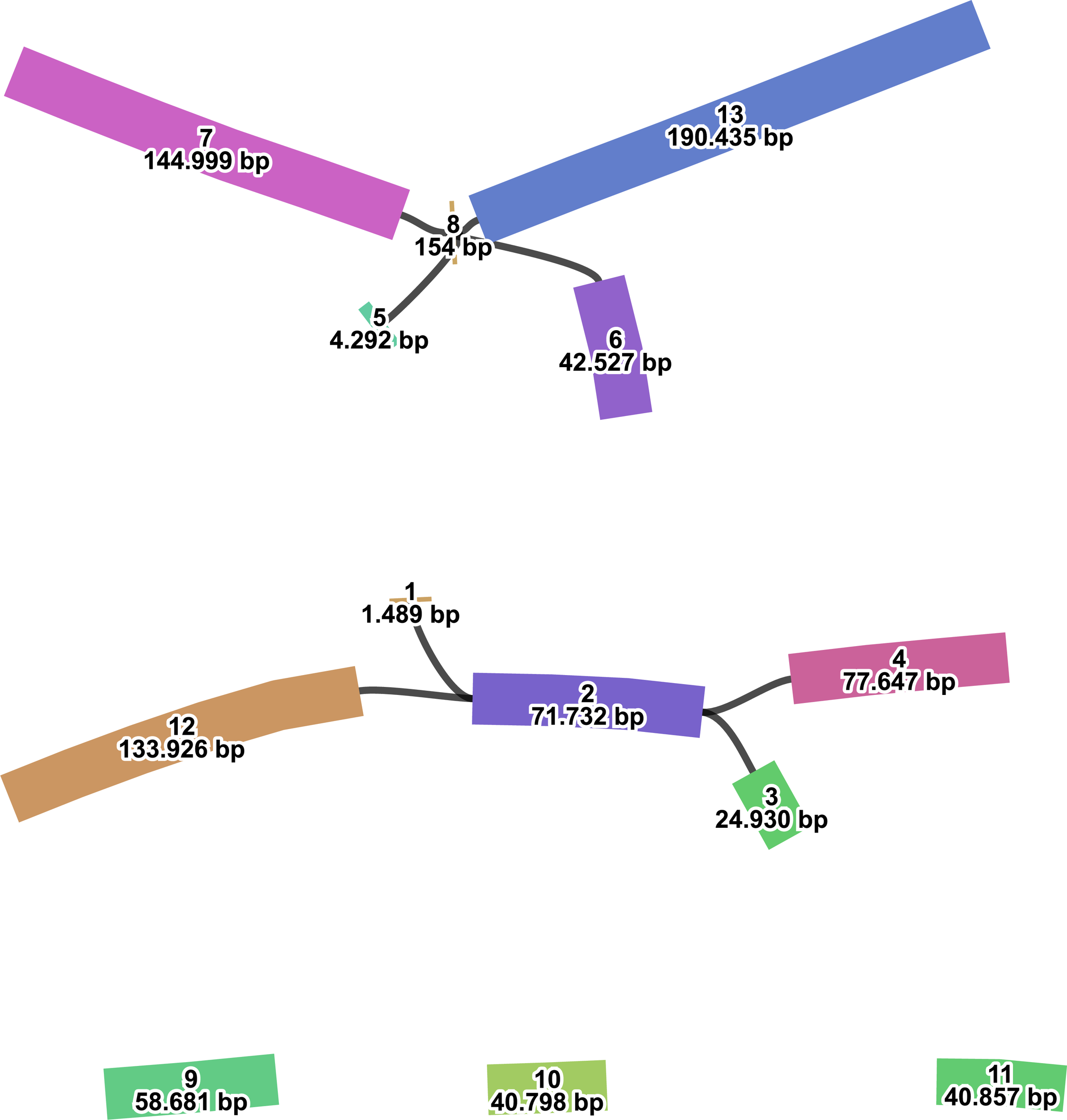

Supplement: S1 Fig — The assembled contigs (nodes, represented as colored bars) with multiple inputs and outputs, and dead ends; and the connections between those contigs (edges, represented as black connectors) are shown. (TIF) [file pone.0180484.s001.tif]

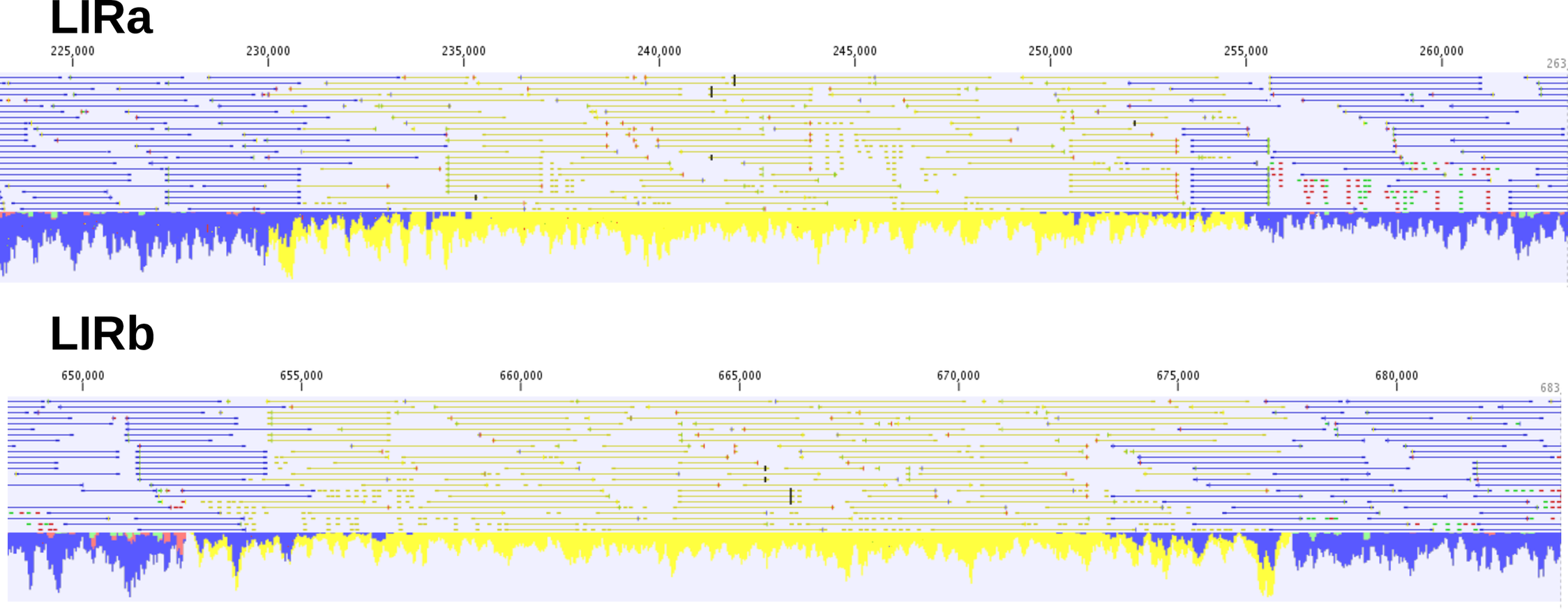

Supplement: S2 Fig — Blue lines represent the paired reads located on the border of the repeat region; the yellow lines represent the paired reads located on each repeated region. Mismatches between the reads and reference are shown as narrow vertical traits. The read coverage are shown as peaks located in the bottom of each figure, whereas blue represent the repeat borders and yellow the repeat itself. (TIF) [file pone.0180484.s002.tif]
